# Prevention of Parkinson’s disease-related sudden death

**DOI:** 10.6061/clinics/2021/e3266

**Published:** 2021-08-23

**Authors:** Fulvio A. Scorza, Antonio Carlos G. de Almeida, Carla A. Scorza, Josef Finsterer

**Affiliations:** I Disciplina de Neurociencia, Escola Paulista de Medicina/Universidade Federal de Sao Paulo (EPM/UNIFESP), Sao Paulo, SP, BR.; II Laboratorio de Neurociencia Experimental e Computacional, Departamento de Engenharia de Biossistemas, Universidade Federal de Sao Joao del-Rei (UFSJ), Sao Joao Del Rei, MG, BR.; III Krankenanstalt Rudolfstiftung, Messerli Institute, Vienna, Austria.

The year 2021 marks 204 years since James Parkinson published his booklet entitled, “An Essay on the Shaking Palsy,” containing the first clear clinical description of the shaking palsy or paralysis agitans, now referred to as Parkinson's disease (PD). However, he was unable to foresee the current illuminating period in PD research (1-5).

PD is the second most frequent neurodegenerative disorder of aging after Alzheimer's disease and the most frequent movement disorder (4-7). In most textbooks, PD is defined as a complex progressive neurodegenerative disorder clinically characterized by tremor, rigidity, bradykinesia/akinesia, and postural instability owing to progressive neuronal loss in the *substantia nigra pars compacta* (SNpc). Additionally, the clinical presentation includes other motor and non-motor symptoms (4,5,8-10). The movement abnormalities in PD are due to the loss of dopaminergic neurons of the SNpc and widespread intracellular aggregates of α-synuclein (4,5,8). Epidemiologically, PD occurs in 0.3% of the general population, in 1.0% individuals older than 60 years, and in 3% of those aged above 80 years, with incidence rates ranging from 8 to 18 per 100,000 person-years (4,5,9). Additionally, the prevalence of PD is estimated to increase by more than 50% by 2030, and the only proven risk factor for the disease is advancing age (4,8,9,11,12). Although there is no known cure for PD, available therapies affect disease symptoms and predominantly focus on the dopaminergic pathway (4,8,11). Correspondingly, potential brain surgery interventions (deep brain stimulation of subthalamic nuclei) for those developing intractable levodopa-related motor complications are successfully applied today (4,5).

Considering these data, it is clear that PD is a progressive, incurable, and systemic neurodegenerative disorder (4,5). Importantly, there is an emerging consensus that cardiovascular disease in patients with PD imposes a considerable burden on mortality, causing the scientific community to be increasingly alert to these real events (4,13). Moreover, several epidemiological data over the years have shown that PD is not a benign condition as individuals with PD have a higher rate of premature death compared to the general population (4,5,7,14-16). More precisely, mortality in PD does not increase in the first 5 years after disease onset, but increases thereafter, with a relative risk of 3.5 after 10 years (4,5). Thus, determining factors, such as aspiration pneumonia, and cerebrovascular and cardiovascular diseases, typically cause deaths in patients with PD (4,17). Additionally, sudden unexpected death in PD (SUDPAR) is considered an important cause of death in PD (4,7). The pioneer authors defined SUDPAR as sudden death in a patient with PD without any satisfactory elucidation of death as determined by autopsy studies (4,7). To date, there are no epidemiological studies that precisely demonstrate the possible incidence of SUDPAR in the leading research centers for movement disorders (4,7). However, a general analysis of relevant studies on SUDPAR since the 70s has shown that an average of 14% of PD individuals die suddenly (4,7). Recent research suggests that multiple risk factors may contribute to SUDPAR, such as age at onset, duration of PD, sex, severity of motor abnormalities, concomitant cardiac and pulmonary disease, drug treatment (polypharmacy), and sleep disorders/circadian alterations (4,7,18). The major domains of potential mechanisms of SUDPAR are autonomic, that is, cardiovascular (4,7,18). Recent data show that cardiac abnormalities and autonomic dysfunction play an important role in SUDPAR, as approximately 60% of patients with PD have cardiovascular disturbances owing to the frequent autonomic disorders in PD (4,7,18).

In general, SUDPAR is a fatal complication of PD. Furthermore, a fundamental practical problem in studying SUDPAR risk factors, mechanisms, and prevention is that it is relatively uncommon. In this context, it would be interesting to develop preventive studies in patients with PD to minimize risk factors for premature mortality (4,7,18,19). Following this line of reasoning, some general recommendations deserve to be emphasized.

- **SUDPAR education targeted at patients, family members, and caregivers:** There is an educational problem concerning mortality in PD as one of the most frequently asked questions about PD is: can patients die from PD? (20,21). The first reaction to sudden and unexpected death is complete disbelief. Thus, imparting education related to SUDPAR is an arduous and complicated task; however, it is the only way to provide access to human emancipation and social transformation (22). Thus, as the discussion of the impacts of premature mortality in a familial context provides best practices in prevention and recovery strategies, it would be appropriate to establish a task force that discusses issues related to SUDPAR to explore these questions.
- **The convergence of clinical healthcare:** Family physicians and other medical and health professionals should develop close collaboration to assess the state of knowledge about SUDPAR. These multidisciplinary team members should consider the personalized risk for heart disease in PD individuals (4) and establish routine cardiovascular screening (electrocardiogram, Holter-monitoring, and echocardiography) to reduce mortality rates in these patients.
- **Supervision at night:** Sleep disorders, such as rapid eye movement-sleep behavior, are commonly reported in patients with PD (23). Nighttime problems significantly reduce the quality of life and are associated with SUDPAR, requiring prompt recognition and intervention (23,24). An interesting study has underlined the importance of nocturnal supervision and a reliable monitoring system against sudden deaths in epilepsy (25). It has been proposed that nighttime supervision should involve the presence of an individual of normal intelligence and at least 10 years of age in the bedroom, or the use of special precautions (for example, a bed breathing alarm) (26,28). Overall, the multidisciplinary team and family members should discuss whether enhancing nocturnal supervision may lower SUDPAR risk.
- **Adequate hydration for patients with PD:** Water is critical for life. Although the recommendation for sedentary adults is to drink approximately 2 L daily, water is frequently neglected as a dietary constituent (29-31). However, age-related water consumption changes make older people vulnerable to dehydration, which is considered the most common fluid and electrolyte disorder in older adults (29,32,33). Thus, as dehydration is a risk factor for the deterioration of PD, it is strongly recommended that patients with PD drink sufficient water to maintain hydration (29). Considering the findings aforementioned, it has now been proposed that adequate hydration levels should be part of clinical practice guidelines for PD (29).
- **Participation in physical activity and sports:** Physical activity (PA) is beneficial for brain functions, improving memory, and learning in age-related neurodegenerative diseases (34). Moreover, PA is important for brain rehabilitation and remodeling (34). Similarly, PA is increasingly advocated as an adjunct intervention for patients with PD (35). Thus, PA is known to improve many symptoms in patients with PD, including physical and cognitive functional capacities (36). Importantly, PA has the potential to improve the non-motor symptoms (depression, apathy, fatigue, constipation) and secondary complications of immobility (cardiovascular, osteoporosis) in PD (36). Based on these data, it is probable that regular PA (under the direct supervision of trained staff) may attenuate cardiac abnormalities that could predispose to SUDPAR.

Overall, new considerations should be persued and experimental, epidemiological, and clinical studies should be conducted to establish preventive measures for PD and their protective effects on SUDPAR with precision. Meanwhile, caution in patients with SUDPAR continues to be prudent and necessary.
